# How cultural beliefs and rituals may help alleviate grief and despair: a four-dimensional framework

**DOI:** 10.3389/fsoc.2025.1620016

**Published:** 2025-11-07

**Authors:** Huy P. Phan, Bing Hiong Ngu, Si-Chi Chen, Chao-Sheng Hsu

**Affiliations:** 1 School of Education, University of New England, Armidale, NSW, Australia; 2 Department of Education, National Taipei University of Education, Taipei, Taiwan

**Keywords:** ancestor worship, cross-cultural mourning practices, cultural grief rituals, emotional resilience, family and intergenerational bonds, life and death education, transpersonal understanding

## Abstract

This theoretical-conceptual article draws on philosophical discourse, cultural analysis, and personal narrative accounts from our teaching and research in *life and death education*, particularly within higher education settings, to examine the important role of cultural grief rituals in contemporary bereavement practices. Focusing on traditions such as ancestor worship, we explore how such rituals provide structured frameworks for processing grief, fostering emotional resilience, and sustaining meaningful connections with the deceased. We propose that these culturally embedded practices can function as contemporary ‘grief support systems’, offering pedagogical and existential insights for individuals navigating death, loss, and mourning. Through cross-cultural examples (e.g., from East Asian, Indigenous, African diasporic, and Western contexts), we attempt to explain how grief rituals (e.g., the practice of ancestor worship) may foster empathy, promote cultural literacy, and help individuals and communities reframe loss in constructive, inclusive ways. In doing so, the article underscores the importance of integrating cultural heritage and its appreciation into life and death education curricula, fostering more holistic and culturally responsive approaches to the teaching and research of the subject, including grief. Finally, we propose a theoretical model—named as the ‘Four-Dimensional Framework for Understanding Cultural Grief Rituals’—which serves to delineate the emotional, communal, cross-cultural, and spiritual functions of these practices. This proposed framework not only informs future sociocultural research on bereavement but also offers practical pathways for enhancing grief education and support strategies in increasingly diverse and globalized learning environments.

## 1 Introduction

*Death* (Rowe, 1997; Murray et al., 2005; Afolabi, 2014) is an inevitable aspect of life, but its aftermath and the process of mourning differ across cultures. A central question arises in how individuals cope with the grief following the death of a loved one. We argue that culture plays a vital role in shaping perceptions of death and in influencing the emotional and psychological strategies used to process loss (Stroebe and Schut, 1999; Proust, 2024). Beliefs about the ‘afterlife’ (Segal, 2004; Jones, 2016), the continued connection between the living and the deceased, and the broader meaning of life itself may determine how grief is expressed and managed. In many cultures, for example, rituals such as *ancestor worship* (Townsend, 1969; Steadman et al., 1996; Lakos, 2010) may offer comfort by maintaining socioemotional bonds between the generations, thus fostering a sense of continuity (e.g., that death is not final) and helping to manage the mourning and/or the grieving process.

An important line of inquiry we propose in this theoretical-conceptual article involves reframing grief associated with death—not as something inherently negative or fear-inducing, but as a natural and potentially transformative aspect of life. We contend that death and the grief that follows are often perceived through a lens of darkness, morbidity, and negativity. From our perspective, however, it is essential to consider pathways, strategies, and opportunities that can help alleviate the existential anxiety and fear frequently associated with loss and grief (Walter, 1996; Stroebe and Schut, 1999; Proust, 2024), fostering a more balanced and accepting emotional response. In recent years, our work in *life* and *death education* (Fu, 1993; Phan et al., 2020; Lei et al., 2022) has contributed to this discourse by addressing these perceived negativities. Grounded in the principles of scientization and normalization of death and dying, we have developed a conceptual framework that incorporates various theoretical lenses, including a ‘Philosophical lens’ (Phan et al., 2025a; Phan et al., 2025b), for studying and teaching death education. We argue that this innovative framework facilitates a more comprehensive and nuanced understanding of this complex and often misunderstood subject.

### 1.1 Focus of inquiry

An interesting question for consideration is how we can make the study of the totality of death education (Rowe, 1997; Murray et al., 2005; Afolabi, 2014)—including the processes of grief and mourning (Gorer, 1965; DuBose, 1997; Hockey et al., 2001)—engaging and accessible for students and others, while also addressing the possibility of reducing the fear and anxiety surrounding it. How can death be presented as a subject worthy of the same academic rigor as any other? Is it possible to alleviate the fear of death? These questions have led us to consider the following premise: *cultural rituals, beliefs, and practices (e.g., ancestor worship) that shape mourning and the understanding of death are not only essential in alleviating grief but also offer valuable insights for enhancing bereavement support systems in today’s increasingly globalized world.*

Examining grief through a ‘Cultural Lens’ (Phan et al., 2025c; Phan et al., 2025d) is encouraged, as individuals often engage with practices and values different from their own (e.g., participating in traditional rituals while living in a multicultural society where such practices may be unfamiliar or misunderstood). This theoretical-conceptual article draws on our *personal narratives* of teaching and research, alongside the *discourse of philosophical psychology* (Thagard, 2014; Thagard, 2018; Phan et al., 2025a), which emphasizes personal intuition, philosophizing, and logical reasoning, to explore how understandings of death, loss, and grief can be deepened. This philosophical psychology lens complements classical sociological perspectives by adding an individual, meaning-making dimension to the collective and structural insights of theorists such as Durkheim (1912/1995), van Gennep (2019), and Turner (1969). Specifically, our subsequent theoretical overview—drawing on selected sociocultural (e.g., East Asian ancestor worship) and religious (e.g., Hindu) perspectives on death education and grief, and informed by personal narratives and philosophical psychology—culminates in the development of a conceptual model we term the *Four-Dimensional Framework for Understanding Cultural Grief Rituals*. This proposed framework, in tandem with a few issues that we identify in the latter sections of the article, establish grounding for further development. For example:

- How do individuals from culturally traditional backgrounds reconcile personal disbelief or secular values with participation in ancestral mourning rituals?
- In what ways do generational differences within the same cultural community affect perceptions of the value and relevance of grief rituals such as ancestor worship?
- To what extent can digital mourning practices—such as virtual funerals and online memorials—replicate the emotional, communal, and spiritual support traditionally provided by in-person rituals?

The three sample lines of inquiry, we contend, have the potential to deepen our understanding of death and, in particular, how contemporary individuals and communities navigate grief amidst shifting cultural norms, technological change, and diverse personal belief systems. Overall, then, this article does not merely describe cross-cultural grief rituals. Rather, it serves as a conceptual analysis, aiming to map the core dimensions through which these rituals operate to support emotional regulation, promote communal bonding, and provide metaphysical or spiritual meaning in times of loss. Our contribution lies in identifying and systematizing these functions across culturally diverse practices—ranging from Confucian ancestor reverence to *Día de los Muertos* (Haley and Fukuda, 2004; Gutiérrez et al., 2015)—thereby offering a framework that can inform grief education, enhance cultural understanding, support therapeutic practices, and guide further sociological research (Kearl, 1989; Howarth, 2007; Walter, 2015).

## 2 A brief mentioning of death education studies

Before we begin, we wish to acknowledge a reflective and theoretical orientation that underpins our scholarly practice. While this article is conceptual and interdisciplinary in nature—drawing on educational, psychological, and cultural perspectives—we recognize that it does not directly engage with empirical research, particularly in relation to death (Rowe, 1997; Murray et al., 2005; Afolabi, 2014), grief (Gorer, 1965; Wagoner and Brescó de Luna, 2022; Afyonoğlu and Pak Güre, 2024), and bereavement (DuBose, 1997; Hockey et al., 2001). Our intention, however, is not to present data-driven findings, but to offer a theoretically informed framework grounded in philosophical psychology and our personal and professional experiences in life and death education. This reflective approach is central to our pedagogy, where we frequently employ open-ended, philosophical discourse to encourage deep engagement with complex existential themes (e.g., the impermanence of life as a lens for understanding loss, change, and personal growth). For example, in a recently published article in *Transpersonal Psychology Review*, titled ‘Does philosophizing about life and death help an individual overcome the hardship of life’s complexities?’ (Phan et al., 2025d), we adopted a similar open-ended approach to invite readers to reflect on the human condition. In the same vein, the present article offers a conceptual framework intended as a foundation for future empirical exploration and interdisciplinary dialogue.

This section of the article provides a brief theoretical overview of our approach to teaching, researching, and understanding the subject of death (Rowe, 1997; Murray et al., 2005; Afolabi, 2014). This framework highlights the importance of integrating diverse perspectives and methodologies. We also incorporate personal narratives (e.g., teaching experiences) where relevant, using these reflections to enrich the discourse on death education. This approach bridges the personal and cultural, deepens academic analysis, and fosters a nuanced understanding of death as a multifaceted phenomenon. As scholars in the field of life and death education—commonly referred to as *life education* (Chen, 2012; Huang, 2014; Phan et al., 2020) and *death education* (Shu et al., 2023; Zhu et al., 2023; Phan et al., 2024)—we have had the privilege of teaching death studies to both undergraduate and postgraduate students for over a decade. Our work in this area has been shaped by a commitment to not only impart knowledge but also to explore the deeper, often complex philosophical and psychological dimensions of death, loss, and mourning (e.g., what happens to one’s ‘soul’ after death?). We have developed a nuanced understanding of how death is understood, experienced, and ritualized across cultures (e.g., the Taiwanese culture), seeking to engage students with both theoretical frameworks and real-world applications. Our research and teaching continue to evolve as we examine new ways, means, and opportunities to address the pertinent questions that arise at the intersection of life, death, and human experience (Neimeyer, 2001; Neimeyer et al., 2011; Wong et al., 2018).

In a pragmatic sense, death education delves into the cessation of life and other practical, daily-relevant issues that individuals may encounter as they navigate the process of dying or deal with the impact of death. Our teaching includes, for example, addressing the significance of *palliative care* (Li et al., 2021; Bollig and Zelko, 2024), such as the provision of end-of-life care for those facing terminal illness (Bollig et al., 2020; Bollig and Rosenberg, 2023). In this context, we also introduce the foundational work of Elisabeth Kübler-Ross (1969), whose five-stage model of grief—(D)*denial*, (A)*anger*, (B)*bargaining*, (D)*depression*, and (A)*acceptance*—offers a structured perspective on the emotional responses to dying and bereavement. Her model aligns with the *Emotional Dimension* of our Four-Dimensional Framework for Understanding Cultural Grief Rituals (outlined later in the manuscript), supporting our efforts to foster emotional literacy and reflective understanding within life and death education. By situating our framework alongside Kübler-Ross’s (1969) contributions, we offer students a broader lens through which to engage with both the clinical and emotional realities of death.

In addition to facilitating a deeper understanding of the physical and/or ‘real-life’ aspects of death, death education emphasizes the importance of compassionate care, which we refer to as ‘life care’ (Phan et al., 2021). This approach ensures that individuals in their final stages of life receive the support, comfort, and dignity they deserve. Emotional well-being and reverence during this phase are essential for both the individual and their loved ones. Compassionate care promotes peace and fosters social connections, which significantly contribute to their mental and emotional health. One co-author, a resident of Taiwan, has shared his personal experience volunteering in end-of-life care. He makes home visits to provide companionship to individuals nearing the end of life, offering physical comfort alongside emotional and spiritual support. Through these visits, he builds meaningful connections with isolated individuals, many of whom struggle with despair, loneliness, and feelings of abandonment. This volunteer work has deepened his understanding of Taiwanese cultural practices related to death and mourning, especially how these customs promote dignity and strengthen community bonds in life’s final stages. By focusing on these personal aspects, death education seeks to bridge the gap between theoretical knowledge and practical application, preparing individuals as they reach the end of life.

Beyond the practical aspects of death, our teaching and research in death education also explore the cultural and philosophical dimensions of life and death. This non-pragmatic aspect is particularly compelling, as it offers insights into the philosophy of existence, mortality, and what may lie beyond. We teach students how diverse cultures and indigenous groups interpret the meaning of life and approach death, alongside the ways in which different philosophical frameworks shape our understanding of the human condition and the nature of existence. This approach goes beyond theoretical study, encouraging students to reflect on how various epistemological beliefs and cultural traditions—such as concepts of life after death (Jones, 2016; Phan et al., 2024; Phan et al., 2025c)—impact both individual and collective perceptions of death. In contrast to the pragmatic aspects of death education, these cultural and philosophical perspectives serve to normalize or even ‘scientize’ the topics of death, loss, and grief.

Particularly significant is the integration of transpersonal or trans-mystical experiences that are often derived from the process of philosophizing about death (Phan et al., 2025a; Phan et al., 2025b). These experiences, which transcend the ordinary bounds of the human psyche and everyday life, provide individuals with a sense of connection to something greater than themselves within the universe. The exploration of such experiences deepens the understanding of mortality, not only through intellectual engagement but also through spiritual and transcendent dimensions (e.g., is there such a thing as ‘premonition’?). Through these perspectives, students are encouraged to confront and make sense of existential questions that transcend personal grief and loss (e.g., can one interact with a loved one who has passed on?), allowing them to broaden their awareness of human interconnectedness and the nature of the afterlife (Segal, 2004; Jones, 2016). In the following sections, we will further explore how these cultural, philosophical, and transpersonal perspectives inform modern practices surrounding death, as well as the mourning and grieving processes, ultimately emphasizing the profound impact of philosophical reflection on how we understand and navigate life’s most fundamental transitions.

## 3 Cultural beliefs: frames of meaning in grief

Small-group discussions are a central feature of our teaching approach, providing students with space to explore complex themes related to death through dialogue and reflection. Questions such as, “Is the concept of the continuity of life, death, and rebirth logical?” or “How do cultural and religious beliefs shape our understanding of mortality?” encourage students to reflect critically and articulate their assumptions. These conversations build appreciation for diverse worldviews and help students develop philosophical and analytical skills for understanding death and mourning.

Beyond practical considerations like palliative care (Bollig and Bauer, 2021; Li et al., 2021; Zhu et al., 2023), our teaching foregrounds a philosophical lens (Phan et al., 2025c; Phan et al., 2025d), inviting students to consider mortality as a transformative and universal human experience. This includes reflection on transpersonal experiences—for example, a Buddhist monk’s account of altered states of consciousness—which raise questions about the boundaries between life and death. We also introduce students to cultural frameworks that shape understandings of grief and continuity. Confucian traditions (Yao, 2000; Havens, 2013) emphasize ancestor reverence and family continuity, presenting death as a transition rather than a final end. Similarly, rituals such as Día de los Muertos in Mexico^1^^,^^2^ and certain forms of East Asian ancestor worship—presented here as illustrative examples rather than exhaustive representations—highlight ongoing bonds between the living and the dead. These examples show how certain local traditions reflect broader themes of remembrance and continuity, without suggesting that all cultures—or even all groups within the same culture—observe them in the same way.

### 3.1 The role of ‘cultural discourse’

Before proceeding—and building on our earlier footnote—we believe it is important to clarify that this theoretical-conceptual article does not aim to provide an exhaustive survey of global religious traditions. Rather, we have purposively focused on selected cultural (e.g., Confucianism) and religious (e.g., Buddhism) perspectives to illustrate our conceptual framework. As authors working at the intersection of Education and Psychology, our theoretical approach draws on personal narratives (e.g., using a particular pedagogical element), transpersonal reflections, and conceptual synthesis, rather than a strictly sociological or anthropological analysis. We are mindful, of course, that this journal’s scope is rooted in sociological inquiry, and we recognize that some readers may not fully align with the epistemological underpinnings or interdisciplinary methods we employ. Nevertheless, we firmly believe that offering our approach—grounded in educational and psychological engagement with cultural grief rituals—can complement sociological understandings and contribute to a more holistic exploration of bereavement and ritual practice. We contend that engaging in what we term the *cultural discourse* of death, loss, and grief (Phan et al., 2025a; Phan et al., 2025b) is both intellectually enriching and deeply insightful. This discourse recognizes that cultural narratives universally—rooted in the broader cycle of life—may offer meaningful frameworks for understanding mortality and the essence of human existence. Death, while biologically universal (e.g., experienced across all human societies regardless of geography or era), is perceived and processed through diverse cultural lenses shaped by longstanding beliefs, traditions, and philosophies. These narratives often provide individuals and communities with guidance during periods of grief, framing loss as a transformation rather than an end. Our forthcoming personal reflections illustrate the importance of such narratives in both personal and pedagogical contexts.

One or two of the co-authors specialize in teaching Buddhist philosophy (Yeshe and Rinpoche, 1976; Master Sheng Yen, 2010; Chattopadhyay, 2022) and Confucian principles (Havens, 2013; Tam, 2016). Their personal convictions shape their teaching of death through an emphasis on reverence and ancestral veneration. For instance, the Confucian concept of *xiào shùn* (孝顺),^3^^,^^4^ or filial piety (Chow and Chu, 2007; Chen, 2016; Tam, 2016), emphasizes obedience and respect toward one’s elders, extending beyond death. Practices such as maintaining family altars, offering food or incense, and tending to graves reflect an enduring relationship between the living and deceased. One co-author, a long-time resident of Taiwan, actively participates in *Qingming* or Tomb-Sweeping Day^5^^,^^6^^,^^7^—an annual ritual of grave tending and remembrance. He explains that this act of honoring ancestors not only supports grieving within a familial and cultural framework but also fosters continuity, identity, and a lasting connection across generations.

Buddhist teachings (Yeshe and Rinpoche, 1976, Master Sheng Yen, 2010, Chattopadhyay, 2022), which emphasize impermanence as a central concept, offer students an alternative framework for understanding death. Within this worldview, death is seen as part of the karmic cycle of *saṃsāra*—the continuous flow of birth, death, and rebirth (Sarao, 2017, Lama and Chodron, 2019). Although not all students interpret concepts like karma and reincarnation literally, exploring these beliefs can help normalize death by framing it as part of an ongoing cycle. In contrast, a co-author who identifies as Christian introduces a different perspective—one grounded in faith, divine presence, and the promise of eternal life (Ratcliff, 1987; Kourie and Ruthenberg, 2008). Including multiple belief systems in our teaching reflects the diversity of worldviews that influence how people understand death and loss (Neimeyer, 2001; Wong et al., 2018). We view this diversity as a valuable resource for expanding students’ understanding of grief across cultures. Our pedagogical approach integrates these traditions with practices such as mindfulness and contemplative reflection. For example, we use walking meditation not only to cultivate presence, but also to support deeper introspection about mortality and the meaning of life (Phan et al., 2021; Phan et al., 2023). In some Buddhist traditions, meditation is believed to deepen awareness and connect practitioners with spiritual dimensions of experience. Colleagues in Taiwan have described powerful moments of reflection during meditation, including a sense of connection with deceased loved ones (Phan et al., 2025c). While these experiences are subjective and not empirically verifiable, they are meaningful within the cultural and spiritual frameworks in which they occur. They highlight how ritual and contemplation can support grief processing and emotional healing.

In conclusion, cultural narratives surrounding death and grief offer critical frameworks for how societies process mortality. Whether through Confucian filial piety, Buddhist impermanence, or rituals like Día de los Muertos^8^^,^^9^^,^^10^ and Japan’s *Obon*,^11^^,^^12^ these traditions recast death as a transformation, not a finality. They turn grief into remembrance, reinforce familial and communal ties, and promote reverence across generations. Through diverse perspectives, we see that cultural traditions foster healing and respect, offering multifaceted approaches to making sense of mortality. Ultimately, they invite us to view death as an ongoing, dynamic dimension of human experience—one that both reflects and transcends our temporal lives.

### 3.2 Ancestor worship: a ‘universal’ theme

Ancestor worship is a widespread tradition found across many cultures, offering structured ways to honor and maintain connections with the deceased (Steadman et al., 1996; Lakos, 2010). Although expressed through different rituals, it reflects a shared concern with memory, lineage, and spiritual continuity. Practices such as incense offerings, food preparation, and grave tending serve not only to honor the dead but also to support those who are grieving. Our teaching and research explore the philosophical foundations of ancestor worship across various cultural contexts, including Chinese, Japanese, Thai, and Vietnamese traditions. These practices integrate spiritual belief with familial responsibility, emphasizing remembrance and respect for past generations. While not all students identify with these beliefs, they often express interest in understanding how such customs influence grief and mourning.

One co-author teaches at a multicultural Taiwanese institution with students from China, Indonesia, Malaysia, Singapore, Thailand, and Vietnam. This diversity, according to the co-author, offers valuable insights for examining both shared and distinct practices across cultures (e.g., comparing the Qingming Festival in China (Chang, 1988; Feuchtwang, 2021) with the Obon Festival in Japan (Smith, 1974; Reader, 1991; Nelson, 2000)—both of which honor ancestral spirits, yet differ in ritual emphasis, seasonal timing, and modes of community participation). Many of these traditions—shaped by Confucian, Buddhist, and indigenous beliefs—emphasize remembrance, reverence, and enduring relationships between the living and the dead. We expand this view by introducing a *trans-mystical* perspective (Hood, 2001; Bronkhorst, 2022; Phan et al., 2024; Phan et al., 2025d), wherein rituals like lighting incense are not merely symbolic but are experienced as forms of communication with the deceased. For example, a son may kneel before an altar and pray, “I ask you, Father, to guide and protect me during my final exam.” For him, this is not a metaphor—it is a sincere invocation and expression of belief. One co-author, grounded in personal belief, regularly engages in such ‘spirit dialogue’, expressing a deep emotional and spiritual connection with deceased relatives. These experiences reveal how ancestor worship serves not only as a cultural formality but also as a source of comfort, guidance, and enduring presence.

Beyond their symbolic and psychological functions, these rituals help alleviate grief by affirming continuity and connection. When a person lights incense for a deceased parent, the act becomes a reaffirmation of the parent’s ongoing role in their life—spiritually or otherwise. In doing so, the ritual affirms that death is not a definitive end but part of an ongoing cycle bridging the known and unknown (Segal, 2004; Jones, 2016). Grounded in spiritual traditions, such acts provide emotional reassurance and help individuals process loss within culturally meaningful frameworks.

While our primary focus has been on East Asian traditions, our research also acknowledges practices from other cultural contexts. For instance, Día de los Muertos (Day of the Dead) in Mexico (Haley and Fukuda, 2004; Gutiérrez et al., 2015) is included here as an illustrative case to demonstrate how a culturally specific practice can transform grief into celebration (see text footnotes 8, 9, and 10). Deeply rooted in pre-Columbian and Catholic traditions, this festival features home altars adorned with photos, mementos, favorite foods, and marigolds—welcoming the spirits of loved ones in joy rather than sorrow. Unlike the reflective tone of the Chinese Qingming festival (Chang, 1988, Feuchtwang, 2021), Día de los Muertos normalizes the coexistence of life and death, fostering healing and strengthening community ties. Similarly, Japan’s Obon festival (Smith, 1974, Reader, 1991, Nelson, 2000) is built on the belief that ancestral spirits return to visit their families (see text footnotes 11 and 12). Families clean graves, offer food, and display photographs to honor their ancestors. The festival’s hallmark, the *Bon Odori* dance,^13^^,^^14^ blends celebration with reflection, symbolizing joy in reunion. Although traditionally observed, Obon has adapted to modern life, with virtual celebrations now emerging—yet the core remains intact: reaffirming bonds with the departed and integrating grief into a culturally resonant cycle of remembrance.

We also acknowledge ongoing debate surrounding the tone of ancestor worship rituals. Should they be solemn and reverent, or celebratory and joyful? Among our team, those with firsthand ritual experience view these practices as inherently serious—akin to funerals—rooted in introspection, gratitude, and respect. The formality and seriousness are seen as essential expressions of reverence. However, celebratory interpretations—while less traditional—offer another path: emphasizing joy, memory, and continued love. These alternative expressions, particularly within diasporic or hybrid cultural settings, may appeal to those seeking to honor the dead in ways aligned with contemporary values. This dichotomy invites deeper questions: Does solemnity inherently express greater respect, or can celebration do the same in a different mode? How might globalization reshape ritual forms while preserving their emotional and spiritual significance? To broaden readers’ understanding, we include Table 1, which presents examples of ancestor worship from cultural groups such as the Yoruba of Nigeria. These snapshots illustrate how different societies approach mourning, remembrance, and spiritual continuity—revealing the diverse ways rituals support the grief process.

**Table 1:** Summary of cultural rituals.

Cultural group	Description
Andean indigenous peoples (Quechua, Peru)	Among the Quechua and other Andean groups^a^^,^^b^, ancestors are seen as spiritual custodians of the land, communities, and agricultural cycles. Rituals, often aligned with key farming seasons, involve offerings of coca leaves, food, and chicha (a traditional fermented beverage). Celebratory ceremonies include music and dance, symbolizing gratitude and the belief that ancestors support agricultural success. These practices intertwine spirituality with practical concerns, reinforcing the idea that ancestors continue to sustain the living through their connection to the land. For instance, during festivals such as Dia de Todos Santos, families bring food and drink to cemeteries, creating an opportunity for reflection and communal bonds.
Celtic Traditions (Samhain):	Celtic festivals like Samhain^c^^,^^d^, marking the end of harvest, focus on the connection between the living and the dead. During this time, it is believed that the veil between the worlds is thin, allowing spirits to visit. Bonfires, offerings of food, and storytelling serve to honor ancestors and secure their blessings. The rituals blend reverence with reflection, emphasizing the cyclical nature of life and the continued role of ancestors in protecting and guiding their descendants.
Hinduism (India):	Hindu traditions emphasize the continuous bond between the living and the dead. During Pitrpakṣa, families perform the Śrāddha ritual^e^^,^^f^^,^^g^, which involves offering food, water, and prayers to deceased ancestors to aid their journey in the afterlife and ensure their blessings on the family. Special mantras and rituals performed by priests help ensure the ancestral spirits’ contentment, strengthening familial karma. These practices underline the Hindu philosophy that one’s lineage is central to spiritual growth and continuity, emphasizing duty (dharma) toward one’s forebears.
Hmong (Southeast Asia):	The Hmong people’s ancestor worship^h^^,^^i^ is integral to their spiritual beliefs, with altars in homes playing a central role. Family members present offerings of food, incense, and symbolic items to gain blessings and protection from their ancestors. The Hmong perceive a reciprocal relationship, where fulfilling ancestor-related duties ensures the spirits’ support and keeps misfortune at bay. Rituals are deeply tied to the cycle of life and death, often conducted by shamans or elders, reaffirming the ongoing connection between generations.
Native American Tribes (e.g., Hopi, Navajo):	Ancestor worship among Native American tribes, such as the Hopi and Navajo^j^^,^^k^^,^^l^, emphasizes the enduring spiritual guidance of the departed. For the Hopi, seasonal ceremonies involving kachina spirits symbolize a connection between the physical and spiritual realms, underscoring the belief in ancestors as active participants in the lives of the living. Similarly, the Navajo hold that ancestors influence and guide the living through spiritual ties, ensuring harmony and protection. Rituals, songs, and storytelling play a vital role in honoring those who came before, fostering a profound respect for the land, community, and ancestral heritage. These practices cultivate wisdom, balance, and a stronger bond with the natural world, enriching communal and spiritual well-being.
Polynesian Cultures:	In Polynesia, ancestors are revered as sources of mana (i.e., spiritual power)^m^^,^^n^^,^^o^, integral to community strength and individual identity. Across islands like Samoa, Tahiti, and Hawaii, rituals include storytelling, offerings, and chants that honor ancestral wisdom and bravery. Burial sites are considered sacred, often located close to family homes to signify the ongoing presence of the departed. This connection underscores the belief that ancestors influence daily life, from guiding decision-making to protecting the community, and that honoring them secures spiritual harmony for the living.
Slavic Traditions (e.g., Dziady):	In Slavic cultures, the Dziady tradition^p^^,^^q^^,^^r^ celebrates and honors ancestors through communal feasts and rituals. Families light candles, prepare special meals, and pray to invite the spirits of the departed. Cemeteries and sacred spaces become sites for reflection and connection, with the rituals aimed at fostering harmony between the living and the dead. The customs emphasize respect and gratitude, ensuring the ancestors’ guidance and blessings remain integral to the community’s welfare.
Yoruba (Nigeria)	The Yoruba people of Nigeria^s^^,^^t^^,^^u^ view ancestors as intermediaries between the spiritual and physical realms, acting as protectors and advisors for the living. Rituals often include, for example, prayers, libations, and offerings such as food or drink to honor the ancestors. Festivals like Egungun celebrate ancestral spirits through masquerades and communal gatherings, where performers in elaborate costumes embody the spirits of the departed. These ceremonies emphasize maintaining harmony between the living and the spiritual world, with the belief that ancestors actively influence the well-being of their descendants by bestowing guidance, prosperity, and protection.

a https://link.springer.com/referenceworkentry/10.1007/978-3-319-27078-4_65

b https://notreaffaireatous.org/the-andean-cosmovision-as-a-philosophical-foundation-of-the-rights-of-nature/

c https://www.ccsna.org/samhain-halloween-and-the-day-of-the-dead

d https://druidry.org/druid-way/teaching-and-practice/druid-festivals/samhain-festival

e https://www.britannica.com/topic/shraddha

f http://mahavidya.ca/2015/06/26/sraddha-death-rituals-and-ancestral-rites/

g https://www.wisdomlib.org/hinduism/essay/markandeya-purana-study/d/doc1121535.html

h https://ethnomed.org/culture/hmong/

i https://religionsmn.carleton.edu/exhibits/show/hmong-religiosity/hmong-shamanism/what-is-hmong-shamanism

j https://www.britannica.com/topic/Navajo-people

k https://www.jstor.org/stable/533862?seq=1

l https://owlcation.com/social-sciences/history-of-native-american-indian-crafts

m https://www.britannica.com/place/Polynesia/Religion

n https://www.britannica.com/topic/mana-Polynesian-and-Melanesian-religion

o https://gaze-tta.com/2024/12/polynesian-tribes-a-tapestry-of-beliefs-and-practices

p https://yourrootsinpoland.com/dziady-supernatural-genealogy/

q https://witia.squarespace.com/blogeng/dziady-and-the-ritual-slavic-masks

r https://postcardpoland.com/culture/dziady-the-slavic-celebration-of-the-dead/

s https://www.biblicaltraining.org/learn/foundations/wm241-essentials-of-african-traditional-religions/wm241-03-tier-3-the-role-of-ancestors-in-the-yoruba-cosmology

t https://digitalcommons.andrews.edu/cgi/viewcontent.cgi?article=1056&context=jams

u https://oxfordre.com/africanhistory/display/10.1093/acrefore/9780190277734.001.0001/acrefore-9780190277734-e-1556

In conclusion, ancestor worship—though practiced in culturally specific ways—shares core themes: *reverence for the deceased*, the *continuation of familial bonds*, and a *belief in the enduring presence of ancestors*. Whether expressed through incense and food offerings, dance, prayer, or storytelling, these practices may offer a bridge between the living and the dead. Across traditions—from East Asia to Africa to Latin America—rituals help transform grief into remembrance and loss into connection. They affirm that death is not final but part of a continuing spiritual journey, ensuring that ancestors remain vital to the cultural, emotional, and spiritual life of the community.

## 4 Conceptualization: cross-cultural understanding and cultural rituals as healing practices

The preceding sections have illustrated the rich cultural diversity of death rituals, highlighting both distinct practices and shared symbolic threads. While customs such as lighting incense, preparing food offerings, or holding communal ceremonies vary across cultures, they converge around universal themes: reverence, remembrance, and the enduring bond between the living and the deceased. Rituals like Día de los Muertos (see text footnotes 8, 9, and 10) exemplify how, across cultural contexts, such practices fulfill the human need for closure, continuity, and connection between past and present.

We concluded the previous section by addressing a central question: should ancestor worship be solemn and serious, or celebratory and joyful? While our collective view tends toward solemnity—shaped in part by co-authors grounded in Buddhist and Confucian traditions—we acknowledge that this preference is culturally and personally informed. What matters most, and serves as the foundation of our conceptualization, is not the tone of ritual but its function:

*Cultural rituals, including ancestor worship, offer structured pathways through which individuals and communities navigate grief, honor the deceased, and restore a sense of meaning and connection in the face of loss*.

These practices—regardless of mood or expression—are culturally and emotionally profound, addressing the universal need to understand and live with mortality. The question, ‘Can it be more?’, reflects how cultural rituals motivate our interest and curiosity to expand the scope of death studies. At the heart of our conceptual proposition is the belief that rituals like ancestor worship (Townsend, 1969; Lakos, 2010) may help normalize death, mitigating associated fears by placing mortality within a meaningful cultural narrative. Our framework, as discuss next, suggests that active participation in such rituals yields tangible psychological, emotional, and spiritual benefits. These rituals are not just passive traditions—they are active expressions of cultural identity that may help individuals feel connected to a broader historical and philosophical heritage.

Rituals can serve both practical and symbolic purposes, helping people express emotions while also connecting with spiritual or deeper metaphysical meanings. Through them, individuals engage deeply with existential questions of origin, relationality, and transcendence. A son lighting incense for his deceased father, for instance, is not merely continuing a cultural practice—he is enacting a belief in ongoing connection, seeking guidance, comfort, and affirmation of shared identity. In this way, rituals serve as vehicles for exploring one’s place within a broader continuum of life and death, linking personal grief to collective memory and temporal experience to eternal significance. From this perspective, cultural rituals function as more than ceremonies—they are structured processes of healing, reflection, and transformation. We identify four core roles they fulfill:

- *Emotional and spiritual navigation*: Rituals help individuals and communities process grief by providing emotional structure and spiritual reassurance.
- *Cross-cultural enrichment*: They foster global understanding and appreciation by highlighting both the uniqueness and shared human significance of mourning practices.
- *Communal cohesion*: By transforming grief into a shared cultural expression, rituals reinforce social bonds and collective resilience.
- *Life wisdom and existential insight*: They offer frameworks for contemplating impermanence, mortality, and the values that shape meaningful living.

These roles underscore the capacity of rituals to support introspection, emotional growth, and human connection. Participation in them is not only culturally affirming but also personally transformative. Regardless of their cultural origin, such practices invite individuals to reflect deeply on life, death, and the enduring ties that link generations. In summary, we argue that cultural rituals should not be dismissed as archaic or purely symbolic. Rather, they represent vital, living traditions that help people make sense of loss, affirm identity, and maintain continuity across time. Their transformative potential lies in their ability to bridge personal experience and collective meaning, making them essential to both healing and cultural understanding.

It is interesting to note, as we conclude this section, that one reviewer of an earlier draft recommended greater engagement with sociological or anthropological theories of ritual, secularization, or grief—particularly the work of Becker (1997), Davies (2017), van Gennep (2019), Ariès (1991), Durkheim (1912/1995), Turner (1969), Hertz (1907/2009), Butler (2004), Bauman (1992), and other theorists of modern mourning practices. As authors, we acknowledge the value of such foundational scholarship and have made a concerted effort to acknowledge and situate this manuscript within the broader theoretical discourse of death, ritual, and bereavement studies. At the same time, however, we clarify that this theoretical-conceptual article is designed as a conceptual mapping rather than a theory-driven premise. The primary aim is to explore the cultural dimensions of grief rituals across chosen global traditions—offering a thematic synthesis rather than an exegesis of canonical theorists.

In further response to the reviewer’s recommendation for deeper engagement with sociological, philosophical, and anthropological theories of grief, mourning, and ritual, we have integrated foundational perspectives that conceptually underpin our analysis. While a comprehensive theoretical overview lies beyond the scope and word count limitations of this article, we acknowledge the intellectual importance of these traditions and their relevance to our proposed framework. Table 2 briefly synthesizes the contributions of key theorists—including Durkheim, Turner, Hertz, Ariès, Becker, Butler, and others—to situate our conceptual mapping within a broader scholarly discourse. We acknowledge, as noted by a reviewer of an earlier draft, that our theoretical overview and subsequent proposition (e.g., Table 3) are grounded in our personal narratives of teaching and research in life and death education (e.g., Phan et al., 2020; Phan et al., 2023; Phan et al., 2025a). Our theoretical approach and distinctive perspective, as outlined throughout, stem largely from our engagement with philosophical psychology (Thagard, 2014; Thagard, 2018; Phan et al., 2025b), which emphasizes personal intuition, philosophizing, and logical reasoning in conceptualizing unknown relationships, theoretical concepts, and phenomena (e.g., the possible existence of consciousness beyond death). In the context of this article, philosophical psychology serves to complement rather than replace classical sociological perspectives. Whereas theorists such as Durkheim (1912/1995), van Gennep (2019), and Turner (1969) illuminate the collective, structural, and ritual dimensions of grief (e.g., Table 2), our use of philosophical psychology lens adds an inward, meaning-making layer—attending to how individuals personally interpret, internalize, and transform their experiences of loss within those shared social frameworks. By combining these perspectives, the analysis integrates both the macro-level patterns of cultural practice and the micro-level processes of individual reflection and existential engagement.

**Table 2:** Key theorists on death, mourning, and grief: a comparative summary.

Theorist	Analysis theoretical focus	Main contribution to grief/death studies	Analysis of relevance to grief
Ernest Becker	Existential psychology	Argues that human behavior is driven by death anxiety. Cultural systems and symbolic acts help individuals deny mortality, offering meaning, identity, and symbolic immortality to manage existential fear. Fear of death drives much of human behavior; culture and ego serve as defenses against mortality.	Grief stems from existential anxiety and the repression of death awareness. Grief confronts individuals with mortality. Becker’s ideas explain how rituals, remembrance, and continuing bonds help mourners symbolically overcome death, reaffirm meaning, and restore emotional balance within cultural worldviews threatened by loss.
Douglas J. Davies	Theological anthropology	Studies attitudes to death; blends ritual theory with belief in immortality and memory. Examines death through ritual, belief, and identity, emphasizing how funerary practices symbolically resist mortality, construct meaning, and maintain continuity between the living and the dead across cultures.	Emphasizes the role of belief, emotion, and ritual in processing grief. Views grief as a relational, culturally embedded process. Rituals and symbolic actions help reconstruct identity, sustain emotional bonds, and transform personal sorrow into communal meaning, enabling coping and ongoing connection.
Arnold van Gennep	Anthropology of ritual	Introduces the concept of rites of passage, highlighting how life transitions—especially death—are marked by ritual stages: separation, liminality, and reintegration, helping societies manage change and continuity.	Frames mourning as a structured social transition for both dead and mourners. Grief reflects the liminal phase in van Gennep’s model, where mourners are suspended between loss and renewal. Funerary rites guide this transition, supporting emotional adjustment and social reintegration after bereavement.
Philippe Ariès	Historical sociology	Traces Western attitudes toward death over centuries, revealing shifting cultural norms—from familiar, public deaths to privatized, medicalized ones—highlighting how societies emotionally and socially construct death and mourning.	Shows that grief is historically and culturally shaped. As death became more private and hidden, grief also grew more internalized, affecting how individuals and communities process loss in modern societies.
Émile Durkheim	Sociology of religion	Defines mourning as a collective social duty; emotions are socially regulated. Emphasizes the social functions of death rituals, arguing that collective ceremonies reinforce social cohesion and shared values, helping societies manage the disruption caused by death and maintain stability.	Grief is not individual but expressed through socially sanctioned rituals. Views grief as a social experience shaped by communal rituals that help individuals express sorrow, connect with others, and reaffirm social bonds, thus facilitating emotional healing and collective resilience.
Victor Turner	Symbolic anthropology	Highlights liminality as a transitional phase in death rituals where participants experience transformation and communitas, reshaping social identities amid loss and change.	Mourning occurs in liminal space; rituals bind mourners into shared solidarity. Sees grief as part of a liminal process, where communal mourning rituals facilitate emotional transition, social bonding, and reconstruction of identity, helping individuals and groups adapt to loss.
Robert Hertz	Social anthropology	Analyzes dual funeral systems and symbolic role of death. Emphasizes the social distinction between the ‘body of the dead’ and the ‘social person’, and the collective ceremonies that manage this transition, reinforcing social order and cohesion.	Views grief as a communal process structured by ritual, helping mourners cope with loss by symbolically separating the deceased’s physical absence from their social identity and reaffirming group solidarity.
Judith Butler	Political philosophy	Explores grief through the lens of performativity and mourning’s social norms, highlighting how loss challenges identity and belonging, while questioning whose lives and deaths are publicly recognized and grieved.	Emphasizes that grief is shaped by societal norms, affecting who is mourned and how; mourning can resist dominant narratives, revealing political dimensions in acknowledging loss and loss’s impact on identity.
Zygmunt Bauman	Sociology of modernity	Modernity seeks to deny or manage death through institutions. Examines death and grief within modernity’s fluid, consumerist society, emphasizing how ‘liquid modernity’ disrupts stable identities and communal bonds, altering how individuals experience and express mourning.	Grief becomes privatized and invisible in consumer-driven cultures. Moreover, grief is complicated by modern life’s impermanence and detachment, where transient relationships and fragmented communities challenge traditional mourning practices and the ability to find lasting meaning in loss.

**Table 3:** Conceptual framework—dimensions of cultural grief rituals.

Dimensions	Function in grief processing	Illustrative examples
i. Emotional	Regulates affect, offers symbolic outlets for sorrow (e.g., ritualized weeping or incense burning to express grief).	Qingming ritual (China)
ii. Cross-Cultural	Promotes empathy, pedagogy, and intercultural understanding (e.g., classroom discussion of mourning rituals from different cultures).	University grief education using multicultural examples
iii. Communal	Reinforces shared values, strengthens family/community bonds (e.g., collective altar-building or group meal following a funeral).	Obon Festival (Japan)
iv. Spiritual	Provides existential meaning, reaffirms beliefs in continuity (e.g., chanting for the dead or reciting prayers for reincarnation).	Buddhist saṃsāra

Collectively, we contend that these theoretical contributions (e.g., in Table 2) help support our central claim: that grief rituals are not merely personal or emotional events but are deeply embedded in historical, cultural, symbolic, and social systems. Although we do not apply these theories in a comprehensive or doctrinal fashion, they help inform the underlying nature of our proposed four-dimensional framework: *emotional regulation*, *communal bonding*, *spiritual meaning*, and *intercultural understanding*. We contend that this integrative approach reflects our effort to appreciate theoretical contributions from others while advancing a useful conceptual framework for further scholarship and practice.

### 4.1 The emotional function of rituals

Rituals surrounding death and grief fulfill what we term an *emotional function*, providing structured frameworks for mourners to process sorrow in ways that are both socially recognized and culturally appropriate. These rituals establish norms for expressing grief while creating communal spaces for emotional support. For instance, one co-author, who previously lived and worked in the South Pacific region, observed a distinctive Fijian mourning ritual that blends somber reflection with celebratory elements. This practice not only alleviates the burden of grief but also strengthens socio-emotional bonds among family and friends.^15^^,^^16^ Why is this the case? Amid profound feelings of loss, sadness, and despair, we purport that rituals may perhaps offer guidance and a sense of stability, allowing mourners to navigate their emotions within a culturally endorsed framework. The co-author, in fact, described this ritual as a learning experience, broadening their understanding of grief practices across cultural contexts.

This perspective contrasts sharply with the experiences of two co-authors raised in collectivist cultures (Herrmann-Pillath, 2010; Biddle, 2012; Fatehi et al., 2020), where emotional restraint is culturally prized. In such contexts, overt expressions of grief—such as crying publicly—may be viewed as inappropriate or disruptive to communal harmony. Within these societies, emotional regulation is deeply entwined with ideals of dignity, composure, and social cohesion. In this light, rituals like ancestor worship serve as culturally appropriate and emotionally meaningful practices. They offer a symbolic outlet through which grief can be expressed in forms that align with societal expectations—quiet gestures such as bowing, lighting incense, and preparing offerings. These acts carry emotional weight while maintaining a sense of decorum and continuity with cultural values.

Personal experiences from several co-authors reinforce this perspective. Practices such as grave sweeping, cemetery cleaning, or preparing annual food offerings—often held on anniversaries or traditional memorial days—transcend their ceremonial formality. They become moments of shared mourning and remembrance, strengthening familial bonds and alleviating the isolating effects of grief. Participating in these rituals affirms a sense of belonging and connection, not only with the deceased but also with the living family and community members engaged in collective remembrance. From a trans-mystical perspective (Phan et al., 2021; Phan et al., 2024; Phan et al., 2025c), such rituals also speak to a spiritual continuity that extends beyond physical death. Rather than viewing death as final, these practices affirm the possibility of an enduring bond between the living and the deceased. This ‘metaphysical orientation’—particularly prevalent in many Asian cultures—reframes mortality within a broader spiritual narrative, offering comfort amid loss and easing existential anxiety. Ancestor worship, in this sense, embodies more than symbolic homage; it enacts a belief in relational persistence across temporal boundaries. One co-author, for instance, expressed a deep conviction that his late father remains present—spiritually or in some other unseen form—continuing to guide, witness, and accompany him through life.

### 4.2 Exchange of rituals and cross-cultural appreciation

Globalization has highlighted the importance of cross-cultural exchange, introducing individuals to a wide range of rituals associated with death, grief, and mourning. This exposure fosters deeper appreciation for cultural diversity, encouraging individuals to recognize and value the distinct mourning practices, beliefs, and values of different communities. Such encounters not only broaden understanding of the universal nature of grief but also emphasize the significance of preserving cultural rituals—such as ancestor worship (Townsend, 1969; Lakos, 2010)—that carry profound emotional and transpersonal meaning. Reflecting on our research team’s international collaboration, we recognize the rich variety of perspectives and interpretations surrounding death and its related practices. This diversity is evident in the team’s personal beliefs and lived experiences. For instance, one co-author’s Christian worldview centers on spiritual salvation, contrasting with another’s commitment to ancestor worship, where rituals before an altar express reverence for deceased relatives. A third co-author holds a philosophical orientation grounded in cosmic continuity, emphasizing the interconnectedness of life beyond physical death. Meanwhile, another co-author, with an agnostic perspective, adheres to empirically validated knowledge and refrains from spiritual speculation. Together, these distinct perspectives demonstrate the complex and multifaceted ways individuals and cultures approach death, grief, and the afterlife—revealing how faith, ritual, and philosophy intertwine in shaping understandings of mortality.

From our standpoint, there is deep richness embedded in the teachings and meanings of cultural rituals associated with death. Practices such as building altars or publicly memorializing the deceased resonate across cultures because they speak to shared human experiences. Far from being mere traditions, these rituals are expressions of reverence and acknowledgment—honoring the universal realities of grief, loss, and mourning, which surpass the ordinary rhythms of life and touch the existential dimensions of being. Through such engagement, individuals and communities access emotional and spiritual connections that affirm the continuity of life and shared humanity. In this context, we contend that embracing the historical and sociocultural diversity of beliefs about life and death is not only valuable but essential. Such openness enriches both personal growth and educational experience, inviting deeper reflection on mortality and existence. By engaging with diverse practices and worldviews, individuals cultivate life wisdom that extends beyond cultural boundaries, reinforcing empathy, understanding, and a more expansive view of human experience.

### 4.3 Importance of community cohesion

Individual grief is a deeply personal response to loss, shaped by one’s emotions and coping mechanisms, often experienced in solitude or within close circles. In contrast, collective grief (Wagoner and Brescó de Luna, 2022; Afyonoğlu and Pak Güre, 2024) is shared, fostering solidarity and healing through communal rituals and support. While individual grief is private, collective grief emphasizes interconnectedness and the universality of loss. Shared experiences—such as the collective mourning following the crash of Jeju Air Flight 2216^17^—forge emotional bonds that lessen isolation and reinforce community resilience.

We contend that cultural rituals play a central role in nurturing social cohesion by creating shared spaces for mourning and collective meaning-making. Practices such as public altars for grieving exemplify how rituals foster mutual support. One co-author, reflecting on her time in collectivist Malaysia, observed how communal care extended beyond kinship, contrasting sharply with her experience in individualistic Australia, where communal engagement in grief was less pronounced (Biddle, 2012). Another co-author, based in Taiwan, shared insights from working with a non-profit offering end-of-life care (Bollig et al., 2020; Bollig and Bauer, 2021). Through home visits and spiritual counseling, particularly the recitation of Buddhist sutras, he observed how rituals fostered a profound sense of ‘family-ness’—a bond that transcends biological connections. He recounts spending hours in the presence of individuals nearing the end of life, sometimes sitting in reflective silence, and at other times engaging in conversations on topics of spirituality, creating a shared space of comfort and connection. This shared sense of belonging provides a space where spiritual wisdom and emotional support intertwine, emphasizing that rituals transcend the act itself; they may indeed function conduits for collective healing and unity.

Building on this understanding, we recognize that collective mourning and active participation in cultural rituals—such as ancestor worship—may play crucial roles in strengthening community cohesion, grounded in solidarity, mutual support, and a profound sense of belonging (Capps, 2003; Baldwin-White et al., 2017; Won et al., 2017). Through shared rituals—whether crafting Día de los Muertos altars (see text footnotes 8, 9, and 10), offering joint prayers, or engaging in ancestral traditions—individuals contribute to a communal expression of grief that affirms the principle that no one should face sorrow alone. These rituals offer opportunities for collective emotional release, reinforcing communal values, and emotional bonds. By fostering collective healing, these rituals help individuals process their grief within a supportive framework, alleviating the emotional weight of loss. Ultimately, these practices strengthen social ties, promoting cohesion and maintaining a sense of connection, even amid profound sorrow.

### 4.4 Role of spiritual wisdom

Our central premise is that *life wisdom*—an embodied understanding of existential realities—enables individuals to grasp the deeper significance of cultural grief rituals, such as ancestor worship (Townsend, 1969; Lakos, 2010). For instance, the Qingming Festival exemplifies how spiritual and transpersonal dimensions are woven into mourning practices, reflecting insights that transcend intellectual knowledge. Unlike content knowledge in fields like algebra or physics (Herscovics and Linchevski, 1994; Star et al., 2015), which is task-specific and compartmentalized, life wisdom encompasses broad, experiential understanding rooted in the human condition. It includes awareness of impermanence, compassion, and the interconnectedness of all beings (Yeshe and Rinpoche, 1976; Thanissaro, 2015; Chattopadhyay, 2022).

We argue that engaging in cultural rituals not only reflects life wisdom but also cultivates it. These rituals address spiritual beliefs in life after death (Ellwood, 2010; Jones, 2016), reincarnation (Nagaraj et al., 2013; Phoenix, 2016), and spiritual continuity (Phan et al., 2025d). Such practices offer existential insight into mortality and relational bonds between the living and the deceased. More than symbolic gestures, rituals like Qingming embody enduring connections across generations and time, offering spiritual continuity that bridges the temporal and eternal. Through these acts, participants internalize cultural narratives, confront life’s impermanence, and develop a richer understanding of universal human values. In this way, grief rituals function not only as comfort mechanisms but also as transformative experiences—deepening personal growth and enriching philosophical reflection. By engaging with their symbolic, spiritual, and communal aspects, individuals cultivate life wisdom that supports both mourning and meaning-making (Neimeyer, 2001; Wong et al., 2018).

### 4.5 Summation

Cultural grief rituals are essential for understanding how individuals and communities process loss and affirm shared humanity. These rituals serve multiple functions—regulating emotion, strengthening community bonds, and exploring spiritual meaning. At the emotional level, they offer culturally grounded structures for expressing sorrow. Through globalization and cross-cultural exchange, such rituals promote appreciation of diverse mourning practices and highlight the universal nature of grief. They also illuminate the power of collective mourning to foster solidarity and communal healing. Importantly, grief rituals cultivate life wisdom—an embodied understanding that bridges personal belief systems with universal existential concerns beyond the reach of conventional frameworks. Our central premise is that participating in cultural rituals offers meaningful support for personal grief, often yielding benefits that extend well beyond the immediate experience. As discussed, these rituals can foster social cohesion, spiritual insight, and emotional resilience, leaving lasting effects on both individuals and communities. From this summative reflection, we propose a guiding structure: the ‘Four-Dimensional Framework for Understanding Cultural Grief Rituals’.

## 5 Contemporary transformations in grief rituals

In many societies, traditional grief rituals (e.g., ancestor worship)—while still practiced—are increasingly mediated, contested, or replaced by emergent alternatives shaped by modernization, secularization, migration, digital communication, and shifting generational values. These factors complicate the continuity of ritual practices and raise important questions about how grief is expressed, shared, and recognized in contemporary life. Modernization and secularization, particularly in Western and urbanized contexts, for example, have diminished the visibility of public mourning (e.g., the decline of traditional funeral processions) and eroded the communal ritual structures that once anchored grief. As a result of the fragmentation of ritual space, some bereaved individuals and their families may experience emotional disconnection or social isolation. In other contexts, likewise, traditional rites—such as ancestor worship, symbolic offerings, or extended mourning periods—may be perceived by younger generations as being outdated or incompatible with modern lifestyles (e.g., younger family members opting for minimal cremation services over customary multi-day mourning rituals). This has created intergenerational tensions, as ritual knowledge may not be transmitted or embodied in the same way.

At the same time, digital mourning practices have emerged as alternative or supplementary modes of grieving (Giaxoglou and Döveling, 2018; Keskinen, 2023; Ai et al., 2025; Al Sheikh, 2025). Online memorial pages (e.g., Facebook), livestreamed funerals, social media tributes, and virtual candlelight vigils, for example, increasingly shape the affective and communal contours of bereavement (Brubaker et al., 2013; Lingel, 2013; Moore et al., 2019; Uwalaka, 2023). These digital rituals expand the space of grief beyond physical locality, allowing diasporic communities or estranged family members to participate remotely. For example, one co-author who was overseas at the time of a relative’s sudden passing participated remotely via a funeral livestream, supplementing this by posting a personal tribute message and lighting a candle at home during the service. While unable to attend in person, these actions nonetheless provided her with a meaningful sense of ‘presence’ and emotional connection, illustrating how digital platforms can help sustain social bonds and facilitate participation across spatial and temporal boundaries.

Nevertheless, the use of digital and hybrid platforms for mourning raises broader considerations regarding *authenticity*, *ritual meaning*, and *emotional presence*. Is it really the same? Can a digital approach (e.g., participation via Facebook) provide an experience equivalent to that of in-person rituals (e.g., grave sweeping)? Does the context alter the emotional resonance of the act? How do participants themselves perceive the value and meaning of online mourning? We posit that online memorial spaces are sometimes shaped more by performative behavior or the design of the platforms—such as algorithms, visibility metrics, or commercial features (Lambert et al., 2018; Jacobsen, 2020; Cheong, 2024; Davoudi and Douglas, 2025)—than by the actual intentions or experiences of mourners. In other words, although digital mourning practices can be deeply meaningful for individuals and families and provide important emotional and social support (e.g., as is the case for one of the co-authors), expressions of grief in highly mediated online environments may at times be influenced by platform norms or reshaped for a wider audience. Indeed, we contend that it is plausible that this discourse or reservation about ‘digital mourning practices’ may have implications with how one interprets, understands, engages in, and remembers grief. By all accounts, we are not seeking to offer a comprehensive critique of digital mourning. Rather, our premise is to underscore the importance of recognizing that online spaces for grief are socio-technical environments with their own affordances, constraints, and cultural expectations.

Seen in this light, digital mourning can be understood both as an extension of traditional rituals—enabling participation, connection, and remembrance across distances—and as a site of ongoing change, where the symbolic and emotional aspects of grief are continually expressed and negotiated. While death and grief remain complex experiences, we argue that rituals are not disappearing but evolving, for example through digital platforms for mourning and memorialization. In multicultural societies, rituals are often adapted to reflect diverse values, such as secularized ancestor altars, interfaith funeral services, or eco-conscious burials. In educational and therapeutic contexts, grief education increasingly incorporates emerging technologies, including AI systems (Hollanek and Nowaczyk-Basińska, 2024; Pillay, 2025; Saraiva, 2025) and other technologies (e.g., virtual reality)(Barberia et al., 2018; Wong et al., 2023; Fanti Rovetta and Valentini, 2025). Adoption is greater in regions with advanced technological infrastructure and more limited where access is restricted. Although the outward form of rituals changes, their *core purposes*—supporting emotional regulation, reaffirming social bonds, and reimagining continuity—remain relatively the same, ensuring grief practices remain adaptive, drawing on both inherited traditions and contemporary innovations to meet the existential needs of an increasingly complex world. In the following section, we provide an overview of our proposed conceptual framework, which may help educators, practitioners, and researchers better understand and navigate the evolving scope of grief rituals.

## 6 Four-dimensional framework for understanding cultural grief rituals

**Figure 1:**
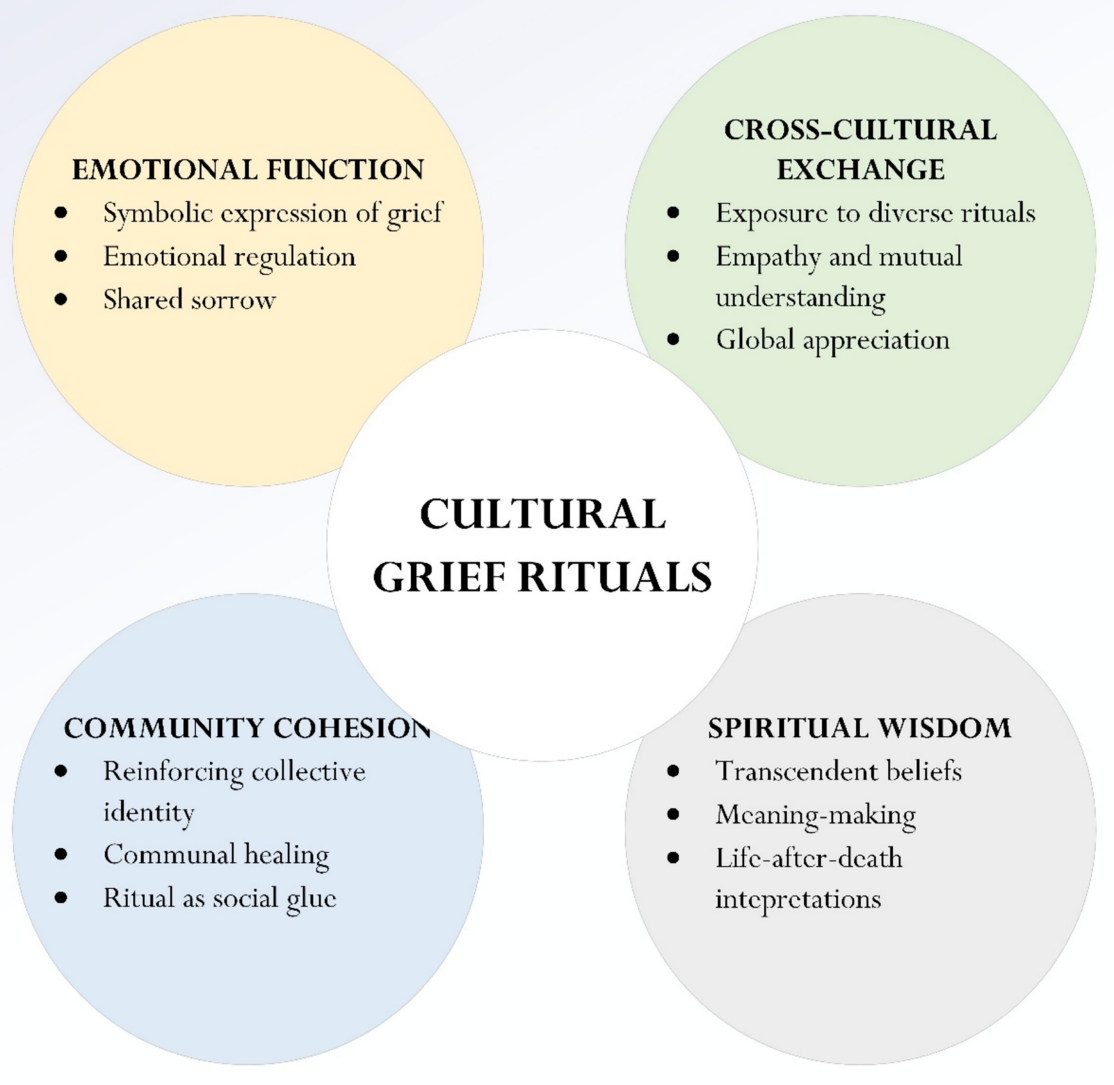
Proposed framework: the four-dimensional framework for understanding cultural grief rituals.

Building upon the thematic and analytical groundwork established in the preceding sections (e.g., Section 4), we propose a conceptual model—named as the Four-Dimensional Framework for Understanding Cultural Grief Rituals—to capture the multi-functionality and evolving relevance of grief rituals across diverse cultural contexts. Ultimately, one could sum up and ask the following: does engagement with and/or appreciation for cultural rituals serve any meaningful role? In the preceding section (Section 4), we intentionally identified, synthesized, and reviewed four distinct premises or functions of rituals, which serve as the basis for developing a theoretical framework of grief ritual practice, clarifying how such rituals operate across emotional, communal, cross-cultural, and spiritual dimensions. By synthesizing these dimensions, the model offers a structured yet flexible lens through which to analyze both traditional and emerging mourning practices. The four dimensions are outlined in Figure 1 and Table 3.

i. Dimension 1: The Emotional Function of Rituals

Cultural grief rituals provide emotional frameworks for navigating the profound and often overwhelming feelings of loss. These frameworks guide individuals in processing grief in ways that align with their cultural and social expectations. They also promote communal spaces for shared sorrow and emotional support, fostering connections among families, friends, and communities. Through symbolic actions and shared activities, rituals can alleviate existential fears and reframe grief within broader spiritual and trans-mystical contexts. A research question that could be asked: *How do cultural grief rituals facilitate emotional regulation and collective healing within diverse sociocultural settings?*

ii. Dimension 2: Exchange of Rituals and Cross-Cultural Appreciation

Global interactions have brought diverse grief and mourning traditions into focus, fostering cultural understanding and mutual appreciation. Exposure to and engagement with such rituals challenge individuals to move beyond personal belief systems, cultivating empathy and respect for diverse approaches to mortality. The diversity in practices, from Christian beliefs in salvation to ancestor worship and cosmological philosophies, underscores the universal nature of grief while celebrating cultural specificity. A research question that could be asked: *How do cross-cultural exchanges of grief rituals foster empathy, mutual understanding, and appreciation for diverse mourning traditions?*

iii. Dimension 3: Importance of Community Cohesion

While grief is often a personal experience, rituals emphasizing shared mourning foster social cohesion. These rituals create a sense of solidarity and mutual support within communities, reinforcing values of interconnectedness and collective healing. In collectivist societies, grief rituals are particularly effective in maintaining communal bonds and ensuring that individuals do not grieve in isolation. A research question that could be asked: *How do cultural grief rituals promote community cohesion by transforming individual grief into collective healing and strengthening social resilience?*

iv. Dimension 4: Role of Spiritual Wisdom

The depth of cultural grief rituals extends beyond emotional and communal realms, reflecting profound spiritual wisdom. This wisdom provides individuals with a foundation to comprehend and internalize the significance of transpersonal dimensions, such as life after death, reincarnation, or continuity of spiritual existence. Life wisdom—shaped through introspection and exposure to diverse cultural practices—enables individuals to reconcile their personal beliefs with universal existential concepts. A research question that could be asked: *How does the integration of spiritual and life wisdom influence the interpretation and practice of cultural grief rituals across cultures?*

The integrated framework described above is both analytical and pedagogical, offering a coherent structure for understanding the functions of grief rituals across emotional, communal, cross-cultural, and spiritual dimensions. Our proposed Four-Dimensional Framework (see Figure 1) extends and complements the foundational scholarship summarized in Table 2. While key theorists—such as Becker (1997), Davies (2017), Durkheim (1912/1995), and van Gennep (2019)—have illuminated important facets of death, grief, and mourning, our model offers a distinctive lens by conceptualizing these four functions as interconnected and operating in tandem. This relational perspective allows for a more dynamic and holistic understanding of how grief rituals function across diverse cultural contexts.

We contend that our theoretical framework may serve both pedagogical and analytical roles. Analytically, for example, as shown in Table 3 (specifically, the column titled “Illustrative Examples”), we consider two case studies: the Qingming Festival (China) and Día de los Muertos (Mexico). What makes these culturally distinct rituals noteworthy? From our viewpoint, both rituals may serve to exemplify how grief practices can operate across the framework’s four dimensions. Qingming fulfills the emotional function (Dimension 1) through incense burning and grave-tending (Chang, 1988; Feuchtwang, 2021) allowing restrained yet meaningful expressions of sorrow, particularly within Confucian contexts that value composure and filial piety. Its communal function is seen in shared family activities that reinforce generational bonds. In contrast, Día de los Muertos takes on a more celebratory emotional tone (Haley and Fukuda, 2004; Gutiérrez et al., 2015), transforming grief into joy through storytelling, food, and music. It promotes communal cohesion (Dimension 3) via public festivals and shared altars. Both rituals also support cross-cultural understanding (Dimension 2): Qingming is increasingly practiced by diasporic Chinese communities, while Día de los Muertos features prominently in global education. Finally, both rituals reflect spiritual wisdom (Dimension 4): Qingming emphasizes ancestral continuity, while Día de los Muertos merges Indigenous and Catholic worldviews to frame death as a transition rather than an end. Pedagogically, these examples also serve as valuable teaching tools. They enable students to compare cultural perspectives, challenge their assumptions, and reflect on grief practices beyond their own traditions. In classroom settings, such case studies can facilitate critical discussion, encourage empathetic understanding, and help learners apply abstract theoretical models to lived cultural experiences. In this way, the framework not only guides analysis but also enhances culturally responsive and reflective teaching practices in the field of life and death education.

In addition to its analytical and pedagogical value, we also view our Four-Dimensional Framework as a useful tool for curriculum development—particularly in designing clear learning outcomes (LOs) within life and death education (Fu, 1993; Phan et al., 2020; Lei et al., 2022) for teaching and learning purposes. In this context, each of the framework’s dimensions can be mapped to specific educational goals. For example, the *Emotional Dimension* can inform learning outcomes related to emotional literacy and the development of empathetic responses to grief and loss (e.g., LO: Students should be able to critically evaluate emotional responses to grief within diverse cultural contexts and reflect on their own positionality). The *Communal Dimension* may guide objectives around understanding the social and relational aspects of mourning practices across cultures (e.g., LO: Students should be able to analyze the role of communal rituals in shaping collective memory and sustaining social cohesion in response to death). The *Cross-Cultural Dimension* supports outcomes aimed at fostering intercultural competence and global awareness (e.g., LO: Students should be able to synthesize cross-cultural perspectives on grief and construct comparative arguments that address cultural assumptions and differences). Finally, the *Spiritual Dimension* allows for reflective learning outcomes focused on existential questions and the meaning-making processes individuals engage in during grief (e.g., LO: Students should be able to develop and articulate an informed perspective on how spiritual or metaphysical beliefs influence attitudes toward mortality and the afterlife). By aligning these four dimensions with curriculum design, educators can scaffold students’ engagement with complex topics such as mortality, ritual, and cultural identity. This approach ensures that teaching extends beyond the acquisition of knowledge (e.g., why certain cultures engage in ancestor worship) to include the cultivation of self-awareness, critical thinking, and intercultural understanding—core competencies in higher education settings.

Finally, our framework also provides a foundation for future empirical research—for example, exploring which dimensions are most salient in specific sociocultural contexts, and why this may be the case (e.g., could the practice of ancestor worship be better understood—or even revitalized—within Western sociocultural contexts through the lens of our theoretical framework?). More broadly speaking, the model may contribute to the literature on death (Rowe, 1997; Murray et al., 2005; Afolabi, 2014), grief (Gorer, 1965; Wagoner and Brescó de Luna, 2022; Afyonoğlu and Pak Güre, 2024), and bereavement (DuBose, 1997; Hockey et al., 2001) by drawing on insights from anthropology, religious studies, psychology, and education to theorize how ritual functions—and understandings of the relationship between life and death—adapt across cultures and contexts. In doing so, it supports the advancement of culturally responsive grief education, therapeutic practice, and cross-cultural dialogue. Ultimately, this framework serves not only as a consolidation of prior scholarship but also as a foundation for future innovation in how human societies ritualize loss in an increasingly complex world.

## 7 Limitations and challenges for consideration

While the four themes and research questions provide valuable insights into the role of cultural rituals in navigating loss, grief, and fostering connections, it is important to recognize a few notable limitations in the existing discourse. Much of the research so far has focused on specific sociocultural contexts, which may not fully capture the range of life experiences across diverse cultural traditions. Additionally, interdisciplinary approaches from sociology, psychology, anthropology, and theology are essential for advancing understanding and addressing gaps in current knowledge of death education (Shu et al., 2023; Zhu et al., 2023). In light of this, it is crucial to examine the evolving dynamics in contemporary societies, which present both challenges and opportunities for maintaining and adapting traditional practices. The following considerations explore how *cultural relevance*, *modern adaptations*, and *internal diversity within cultures* may shape individual approaches to grief and mourning.

### 7.1 Cultural relevance and applicability

Cultural grief rituals may play a crucial role in offering structure and support during times of death, loss, and grief, often connecting individuals with broader cultural, familial, and spiritual traditions (Hidalgo et al., 2021). As we described, participating in rituals such as ancestor worship (Townsend, 1969; Lakos, 2010) can help individuals deepen their connection to family and heritage. Having said this, though, it is important to acknowledge that not all beliefs, values, and rituals align with every member of a community. How might an agnostic teenager who does not believe in reincarnation (Ellwood, 2010; Nagaraj et al., 2013) or the concept of saṃsāra (Sarao, 2017, Lama and Chodron, 2019), for example, engage with these rituals? This mentioning, we contend, highlights the need to consider individual differences in belief systems and how they may influence one’s participation in culturally significant practices surrounding death and grief.^18^ In such cases, the alignment between an individual’s beliefs and the cultural rituals surrounding death may present challenges, underscoring the need for more inclusive approaches in grief practices.

Even within seemingly homogeneous communities, we acknowledge that individuals’ engagement with cultural practices often varies based on their personal experiences, beliefs, and evolving societal norms (Hidalgo et al., 2021). For example, based on our personal experiences, not everyone may feel inclined to believe in or participate in cultural rituals. This issue becomes increasingly pronounced in the context of modernization, which has contributed to the decline of traditional mourning practices in many societies.^19^^,^^20^ The widespread influence of globalized ideals, a move toward more individualistic ideologies, and the rise of secular worldviews have gradually undermined collective cultural practices that once served as the cornerstone for expressing grief and honoring the deceased. In particular, younger generations in many parts of the world may feel less connected to traditional mourning rituals.^21^ Practices such as ancestor worship, grave visits, and communal mourning ceremonies, which once held great emotional and spiritual significance, have become viewed by some as outdated or irrelevant. From our observations, for instance, older generations in Taiwan have expressed concern about the younger generations’ diminishing reverence for ancestor worship and other ritual practices. This decline in the practice of ancestor worship signifies not only the fading of an important tradition but also a potential loss of cultural identity.

With the advent of technology,^22^ likewise, some individuals may even choose to express their grief online, using social media platforms to share their emotions and memories (Ware, 2016; King and Carter, 2022). We must admit to feeling a degree of ambivalence about leveraging technology to share experiences of grief online. In this analysis, we strongly believe that grief and reverence for the deceased are profoundly sacred and should remain within the intimate context of immediate family. Such profound emotions, we contend, are not suited to being ‘placed’ online for public viewing and commentary. In some cases, these practices have become overshadowed by competing social and cultural forces, including individualistic pursuits (e.g., financial pursuit) or purely pragmatic concerns regarding time, effort, and resources. As traditional rituals lose their relevance or are abandoned altogether, many individuals are left without familiar frameworks to help them navigate the emotional and social challenges posed by grief.

Consequently, we argue that research exploring how contemporary individuals experience loss outside traditional rituals is crucial for understanding the deeper psychological and emotional impacts of the decline of these practices. Such research holds the potential to reveal the challenges people face in the absence of established cultural frameworks for grieving and how this shift influences their emotional well-being. It is equally important for research to address how aspects of these lost or evolving traditions can be adapted or reintegrated into modern, multi-faceted approaches to mourning (e.g., the acceptance of social media?). These new frameworks must remain culturally relevant while resonating with younger generations, who may find traditional customs less accessible or meaningful in a rapidly changing, technology-driven world. By investigating how to reinvigorate these practices, we can offer individuals more engaging and effective ways of processing grief, fostering both emotional healing and a continued connection to their cultural heritage. Is it possible, for instance, that future societies might abandon traditional grief rituals, replacing them with new, innovative practices that eventually become the norm?

### 7.2 Addressing diversity within cultures

Another significant challenge in the study of cultural grief rituals is recognizing the diversity that exists within cultures themselves. While cultures offer broad frameworks for understanding death, grief, and loss, they are far from monolithic (Gire, 2014). Within any given cultural context, there exists a wide range of individual belief systems, values, and personal approaches to grieving and mourning. We argue that this internal diversity may complicate the assumption that there is perhaps a universally accepted way of grieving or understanding the nature of death and loss within a particular group. Acknowledging this nuance is crucial for any comprehensive analysis of cultural grief rituals, as it highlights the need for society to consider the individual variations and personal meanings that influence how people navigate these complex experiences. For example, the belief in ancestor worship can be interpreted in different ways within the same cultural context. One co-author views it primarily as an act of respect and veneration, where individuals honor the deceased as a way to maintain familial and cultural traditions. However, another co-author contends that ancestor worship serves a more interactive purpose, seeing it as a means through which individuals can ‘communicate’ with the dead. This perspective suggests that these rituals are not just about honoring the past, but also about establishing a continued relationship with ancestors, seeking guidance, comfort, or blessings.

As highlighted, among those who practice ancestor worship, attitudes toward the importance of rituals can vary significantly depending on factors such as geographic location, socio-economic class, or personal experience with grief. For some individuals, for example, traditional rites (e.g., lighting incense) are integral to their cultural identity, such as fostering a sense of connection to Taiwanese heritage. These individuals may view the rituals as crucial not only for honoring the deceased but also for maintaining continuity with their cultural traditions. That children are raised with the understanding and belief that ancestor worship is a sacred tradition, and one that should be preserved and passed down through generations. Similarly, dragon dancing, a tradition celebrated worldwide during the Lunar New Year, serves as a vital expression of cultural identity for many Asian cultural groups (e.g., Vietnamese).^23^ In contrast, some individuals may find that cultural practices surrounding death and grief do not align with contemporary societal norms or resonate with their personal beliefs and experiences, particularly if they embrace a more secular or individualized approach to mourning. This diversity underscores the necessity of viewing mourning and grief rituals not as rigid, one-size-fits-all practices but as fluid, evolving customs that can differ greatly from person to person, shaped by personal history, social context, and broader cultural influences.

We contend that it is essential to recognize that generational differences play a significant role in shaping how death is understood and how grief is expressed. Based on our observations and personal experiences, older generations often adhere to culturally rooted practices, considering them essential for honoring tradition and showing respect. Largely, the act of engagement (e.g., ancestor worship) is automated and rooted from deep within, becoming an important part of a person’s cultural identity. One co-author highlights the importance of adhering to specific protocols in ancestor worship rituals, such as the proper method of lighting incense and reciting prayers. For instance, in Taiwanese tradition, it is customary to take three incense sticks, bow three times before lighting them, and then bow another three times after they are lit before placing them in front of the altar or grave. Younger individuals, of course, may reframe or challenge these rituals, influenced by their personal preferences, exposure to new ideologies, and the globalized environment they navigate. Such disaccords—where younger individuals choose to forgo the belief in and/or the practice of cultural rituals (e.g., ancestor worship)—may, in fact, reflect the diminishing priority that these rituals hold in contemporary societies. This pattern is evident in various cultural contexts, where younger generations are less inclined to engage in traditional practices, leading to concerns about the loss of cultural identity and heritage. These observations, such as the younger Taiwanese people’s disregard for traditional rituals, underscore the importance of understanding and addressing the factors (e.g., peer pressure) that may account for the decline in participation of cultural rituals among younger generations to preserve cultural identity and heritage. In Taiwan, for instance, a significant portion of the adult population has shifted away from traditional religious practices. About a fifth of Taiwanese adults (21%) have moved away from Daoism since their childhood, and only 24% currently identify as Daoist, down from 42% who were raised in the tradition. This shift indicates a broader trend of younger generations distancing themselves from cultural rituals, raising concerns about the preservation of cultural identity and heritage. We contend that future research could explore how individuals within the same cultural group (e.g., Muslims in the Indonesian historical-sociocultural context) negotiate their diverse beliefs and preferences regarding grief. Such an exploration could foster greater inclusivity in ritual design and lead to a deeper understanding of how grief rituals can honor this diversity while preserving cultural significance. Addressing this historical and sociocultural diversity may involve employing research methodologies that incorporate a range of perspectives and experiences of grief within a given cultural framework, ensuring a comprehensive and nuanced analysis.

### 7.3 Bridging cultural traditions and modern practices

A primary challenge in addressing the decline of traditional mourning rituals, as indicated earlier, is bridging the divide between time-honored and cultural practices and the contemporary needs of society. This emphasis highlights the significance of modernity’s impact. In many societies, nowadays, increasing urbanization, technological developments, and time pressures have made it somewhat difficult for families to participate in practices that were considered as the ‘norm’ of the mourning process. For instance, rituals such as visiting ancestral graves or maintaining tombs have long provided a vital space for reflection and collective sorrow. However, with the rise of urbanization and the fragmentation of family structures due to migration or work demands, the act of physically tending to graves or participating in communal burial rites has become logistically challenging. One co-author, currently residing in Taipei City for work and family reasons, shared his struggles with maintaining such practices. For instance, taking time off during the semester to travel to his hometown for the Qingming Festival, a ritual involving cemetery visits and the cleaning of relatives’ graves, proves to be increasingly difficult. Many individuals, like the co-author, no longer live near their ancestral homes, which complicates the continuation of these traditions. This physical separation from the deceased and the rituals surrounding them can lead to feelings of alienation or unresolved grief, underscoring the impact of modern societal shifts on the mourning process. Similarly, another co-author recently faced challenges in traveling home to attend her late brother’s funeral, highlighting the emotional and logistical difficulties of modern living arrangements across geographical distances.

New forms of memorialization have emerged as meaningful outlets for grief, addressing challenges posed by physical distance, societal changes, and modern constraints. These alternatives include virtual ceremonies, digital memorials, and online communities that commemorate the deceased and provide support during mourning. Practices such as virtual funerals, digital altars, and social media memorial pages enable individuals to express sorrow and celebrate life while overcoming geographical and temporal barriers. These approaches have become particularly prevalent since the Covid-19 pandemic, which significantly hindered traditional mourning practices for many. That said, we acknowledge that while technological and social innovations address some of the limitations inherent in urban or modern life, they are not without shortcomings. Questions remain about their emotional efficacy in replicating the depth of traditional practices. For instance, while virtual ceremonies offer convenience and accessibility, do they carry the same spiritual and emotional resonance as in-person rituals? Can they truly foster the collective sense of community and shared empathy that define more traditional settings? A few of us, as noted, feel somewhat ambivalent about using technology as a platform for expressing grief (Ware, 2016; King and Carter, 2022). We believe that grief is a sacred experience that should remain within the intimate context of family.

Research is therefore essential to examine the appropriateness and effectiveness of modern alternatives, such as virtual rituals, in meeting the needs of mourners. Specifically, studies could investigate whether these innovations successfully provide the emotional, transpersonal, and spiritual support traditionally offered by in-person communal mourning. This line of inquiry could explore how different demographics and cultural groups perceive and engage with these alternatives, as well as whether they foster the same sense of connection, closure, and shared empathy that conventional rituals aim to achieve. For example, does virtual discourse provide the same feelings of empathy and understanding as face-to-face interactions during rituals mourning the death of a loved one? Can one foster a state of solidarity using alternative contemporary methods? By delving into the nexus between technological advancements and personal fulfillment, researchers can contribute to the development of best practices for understanding death and mourning rituals that are both culturally adaptive and aligned to contemporary lifestyles. Such research may hold the potential to bridge the gap between tradition and modernity, ensuring that rituals remain meaningful and accessible while respecting the evolving needs and values of a diverse, globalized society. This exploration could also uncover ways to enhance the design of virtual rituals (e.g., using animated agents), integrating elements that amplify their emotional and spiritual resonance, ultimately creating a more holistic mourning experience. In other words, one may wish to consider strategies that integrate and balance both traditional and modern approaches to grieving and mourning. For instance, incorporating technological advancements into ancestral worship rituals can provide new, meaningful avenues for understanding.

### 7.4 Summation

We contend that a theoretical understanding of death, grief, and mourning rituals reveals a paradigm shift in how individuals and their families engage with tradition amid societal change. Urbanization, technological advancement, and shifting cultural norms have disrupted established practices such as Qingming, giving rise to virtual ceremonies and social media memorials. These innovations address logistical and personal barriers, particularly for those distant from traditional mourning spaces. Yet, while offering accessibility, they may not fully replicate the emotional, spiritual, and communal depth of in-person rituals.

Our analysis has explored the potential of these new forms to foster connection and closure while highlighting how cultural and individual beliefs shape ritual engagement. Understanding the intersection of tradition and modernity is vital for developing practices that remain meaningful, inclusive, and responsive to contemporary realities. At the same time, the scope of this framework necessitates certain limitations. Cross-cultural coverage is broad rather than deep, and our theoretical engagement—drawing from sociology, anthropology, philosophy, and death studies (e.g., Table 2)—is selective by design. The rapidly evolving nature of mourning practices under modernization, globalization, and digitalization further underscores the need for empirical research, particularly digital ethnography, to examine how grief is mediated across generational and diasporic contexts.

Cultural translation and pedagogical application pose additional challenges. While our framework seeks to be inclusive, it may not fully capture the ontologies or metaphysical logics embedded in Indigenous, animist, or Non-Western grief traditions (e.g., the ritual of ancestor veneration during *Guan Luo Yin*, which reflects a cosmology in which the dead maintain active relational roles with the living). From our perspective, it is crucial that educators and practitioners remain sensitive to these evolving complexities and avoid universalizing grief experiences. Nonetheless, these limitations do not detract from our proposed framework’s conceptual value (e.g., Table 3). Rather, they signal the need for continued interdisciplinary inquiry that bridges theory and practice, tradition and innovation, and ritual form with emotional function. The tensions we identify are not constraints, but generative points of departure for ongoing research, curriculum development, and culturally responsive bereavement care.

## 8 Conclusion and implications

Overall, this article has presented a conceptual synthesis of death and grief rituals across diverse cultural traditions—including East Asian, Indigenous, African diasporic, Latin American, and Western contexts—to illustrate that mourning, and indeed grief itself, is never solely a private or emotional experience. Rather, grief rituals operate as structured, symbolically mediated, and socially situated practices that may guide emotional healing, preserve ancestral memory, affirm communal identity, and evoke transcendent meaning (e.g., the Obon Festival in Japan, which combines ancestral remembrance with communal celebration and spiritual reflection). Through this examination, we have proposed a four-dimensional conceptual framework—*emotional*, *communal*, *spiritual*, and *intercultural*—that captures the multifaceted functions of ritual in human grieving. Importantly, our synthesis moves beyond the descriptive cataloguing of global traditions. It provides a theoretical lens through which scholars and practitioners can examine how grief is regulated, ritualized, and transformed across social contexts. In an era marked by secularization, generational change, and the digitization of mourning, traditional rituals continue to evolve or fragment. Yet the core functions of grief rituals—namely emotional regulation and social reintegration—remain deeply embedded in human culture. Our proposed framework, or model, may therefore offer a more nuanced understanding of how grief is not only managed within individuals, but also negotiated across families, generations, institutions, and belief systems.

For sociological research, the Four-Dimensional Framework for Understanding Cultural Grief Rituals may, perhaps, serve to encourage renewed attention to ritual as a living, adaptable social form rather than a static tradition—recognizing that such belief (e.g., the idea that traditional mourning rituals are fixed and no longer relevant in modern societies) risks overlooking how rituals evolve to meet changing emotional, cultural, and technological needs. By reconceptualizing grief rituals as evolving and dynamic expressions of collective identity and spirituality, this framework invites inquiry into how such rituals are reinterpreted, contested, or revitalized across different social groups, digital platforms (e.g., online memorial pages), and historical moments. Our theoretical overview, indeed, has bridged classical theories of mourning (e.g., Hertz, 1907/2009; Durkheim, 1912/1995; Turner, 1969; Bauman, 1992) with contemporary realities—such as hybrid funerary practices, online memorialization, and interfaith mourning rituals (Walter et al., 2012; King and Carter, 2022)—by highlighting the continuity of ritual function amid shifting forms. For practice, particularly in death education (Rowe, 1997; Murray et al., 2005; Afolabi, 2014), bereavement counseling, and intercultural pedagogy, the framework offers a culturally responsive and pedagogically applicable lens to help students, professionals, and families better understand the complex terrain of grief in a globalized world.

In conclusion, then, the primary contribution of this article is conceptual: by synthesizing philosophical, cultural, and psychological perspectives, we map four interrelated functions through which cultural rituals support grief—emotional regulation, communal cohesion, spiritual meaning, and intercultural engagement. This integrative framework provides a foundation for empirical inquiry, educational practice, and culturally responsive grief support in a diverse world.

## Footnotes

^1^ https://maxwellmuseum.unm.edu/sites/default/files/public/El%20Dia%20de%20los%20Muertos.pdf

^2^ https://mexic-artemuseum.org/wp-content/uploads/2020/10/Day-of-the-Death-Guide.pdf

^3^ https://www.chinesepod.com/dictionary/%E5%AD%9D

^4^ https://reloproeura.blog/2019/05/21/intercultural-word-of-the-week-xiao-shun/

^5^ https://studycli.org/chinese-holidays/qingming-festival/

^6^ https://www.nytimes.com/2023/03/27/dining/tomb-sweeping-day-food.html

^7^ https://mandarinmatrix.org/origin-of-tomb-sweeping-day-qing-ming-festival/

^8^ https://kids.nationalgeographic.com/celebrations/article/day-of-the-dead

^9^ https://www.history.com/topics/halloween/day-of-the-dead

^10^ https://www.journeymexico.com/blog/day-of-the-dead-in-mexico

^11^ https://www.jrpass.com/blog/obon-festival-celebrating-the-spirits-of-japan

^12^ https://hwpi.harvard.edu/pluralismarchive/news/japanese-festival-dead-obon-world-religion-news

^13^ https://www.inhamamatsu.com/culture/bon-odori.php

^14^ https://allabout-japan.com/en/article/10913/

^15^ https://ffwrfromfiji.wordpress.com/tag/fijian-funeral/

^16^ https://www.vagabondjourney.com/a-traditional-funeral-in-fiji/

^17^ https://www.airlineratings.com/articles/jeju-air-crash-latest-analysis

^18^ https://www.britannica.com/list/7-unique-burial-rituals-across-the-world

^19^ https://theconversation.com/what-ancient-cultures-teach-us-about-grief-mourning-and-continuity-of-life-86199

^20^ https://greatergood.berkeley.edu/article/item/what_happens_when_we_lose_our_social_rituals

^21^ https://www.internationalcollegeofprofessionalcelebrants.org/blog/how-different-generations-deal-with-death

^22^ https://newconnectionspsychology.com.au/blog/grieving-in-the-digital-age-coping-with-loss-in-a-connected-world/

^23^ https://www.chinahighlights.com/travelguide/special-report/chinese-new-year/new-year-dragon-dance.htm
